# Bonding in Mercury-Alkali Molecules: Orbital-driven van der Waals Complexes

**DOI:** 10.3390/ijms9060926

**Published:** 2008-06-02

**Authors:** Elfi Kraka, Dieter Cremer

**Affiliations:** 1 Department of Chemistry, University of the Pacific, 3601 Pacific Avenue, Stockton, CA 95211, USA; 2 Department of Chemistry and Department of Physics, University of the Pacific, 3601 Pacific Avenue, Stockton, CA 95211, USA

**Keywords:** *mercury*-alkali diatomics, mercury-alkali cations, van der Waals complexes, bonding, relativistic effects, akali metal amalgams

## Abstract

The bonding situation in mercury-alkali diatomics HgA (^2^Σ^+^) (A = Li, Na, K, Rb) has been investigated employing the relativistic all-electron metho*d Normalized Elimination of the Small Component* (NESC), CCSD(T), and augmented VTZ basis sets. Although Hg,A interactions are typical of van der Waals complexes, trends in calculated D_e_ values can be explained on the basis of a 3-electron 2-orbital model utilizing calculated ionization potentials and the D_e_ values of HgA^+^(^1^Σ^+^) diatomics. HgA molecules are identified as *orbital-driven van der Waals complexes*. The relevance of results for the understanding of the properties of liquid alkali metal amalgams is discussed.

## 1. Introduction

The concept of the chemical bond is one of the most successful heuristic approaches to understand the structure and stability of molecules [1–11]. Often this concept is used as if the chemical bond is an observable molecular property. This however is not the case because one cannot define an hermitian operator for any bond property (bond length, bond energy, bond polarity, etc.) that would guarantee a direct measurement of these quantities thereby making the chemical bond observable [12]. A rigid definition of the chemical bond is impossible for principal reasons.

Nevertheless models of the chemical bond have enormously stimulated the progress in chemistry during the last 100 years and still have an important impact on modern developments in chemistry [1–11]. Recent developments in bonding theory have especially focused on an improved understanding of bonding in transition metal complexes, for which relativistic effects, exchange interactions, the influence of core and lone pair electrons can play a decisive role [13]. The present work is part of a larger project aimed at explaining the different modes of bonding when one of the partner atoms is mercury.

Mercury possesses a [Xe]4f^14^5d^10^6s^2^ electron configuration and is according to Pauling or Allred-Rochow electronegativities (χ = 2.00; χ = 1.44 [14]; Table 1) electropositive (less electronegative than H [14]). The electronic structure of Hg is characterized by distinct relativistic effects [15,16]. The 6s-orbital is significantly contracted due to the mass-velocity effect, which in consequence causes a slight expansion of the 5d orbitals. This leads to a decrease in the orbital energy gap between 5d and 6s AOs whereas the energy difference between 6s and 6p AOs increases significantly. Excited states of Hg involving the 6p orbitals are high in energy (^3^P: 119.8 kcal/mol; ^1^P: 153.5 kcal/mol [17]), which explains the negligible involvement of its 6p orbitals in bonding. Similarly, spin-orbit coupling is moderate for Hg [18] because larger effects require a fractional occupation of the 6p or 5d AOs. In those cases where unpaired electrons occupy an orbital with zero angular momentum, the influence of spin-orbit coupling effects on the molecular energy should be rather small [19,20].

Mercury bonding is reasonably understood in the case of mercury halides HgX (X = F, Cl, Br, I) [21]. Due to the electronegativity of the halogens, charge is transferred from the 6s(Hg) to the partly occupied *n*p(X) orbital thus establishing a covalent single bond of moderate stability (8 – 31 kcal/mol [22]) that depends on the electronegativity of X and the degree of charge transfer [21].

The question arises whether any residual covalent bonding is established in the case of alkali mercury diatomics, HgA (A = Li, Na, K, Rb). The alkali atoms are more electropositive than Hg and therefore should donate charge to rather than accept charge from Hg. Apart from this, there is the possibility of 3-electron 2-orbital bonding, which can lead to considerable stability (example He_2_^+^, bond dissocation energy BDE = 56.9 kcal/mol [22]) depending on orbital overlap and orbital energies. In this work, we will investigate the nature of bonding in HgA (^2^Σ ^+^) and contrast it with the bonding in the closed shell cations HgA^+^ (^1^Σ ^+^) where in both cases we will consider the influence of scalar relativistic effects on bonding. Our results will be of interest for HgX bonding in general, the formation of gaseous alkali mercury compounds, and the interaction of mercury and alkali in alkali amalgams, which have a variety of technological application possibilities.

## 2. Computational Methods

In view of the strong scalar relativistic effects observed for Hg, we have used all-electron relativistic coupled cluster theory to obtain reliable quantum chemical description of HgA (^2^Σ ^+^) and HgA^+^ (^1^Σ ^+^). Preliminary calculations were carried out with the zeroth order regular approximation with gauge independence (ZORA-GI) method [23] and density functional theory (DFT) with the B3LYP hybrid functional [24–26] to obtain suitable starting geometries. Results were improved by applying the normalized elimination of the small component (NESC) approach [27,28] that presents a more complete method for determining scalar relativistic effects than the regular approximation does [28]. This was done first with B3LYP whereas final results were obtained with CCSD(T), i.e. coupled cluster theory including all single (S) and double (D) excitations and a perturbative treatment of the triple excitations (T) [30]. The magnitude of the relativistic correction was obtained by carrying out non-relativistic calculations at the B3LYP and CCSD(T) levels of theory. In total, five different levels of theory were employed: B3LYP, CCSD(T), ZORA-GI/B3LYP, NESC/B3LYP, and NESC/CCSD(T). Bond length optimizations were carried out at all levels of theory. Open shell dublets were calculated with unrestricted methodology at the UHF-CCSD(T) and UDFT level of theory.

The Hg basis used is a (22s19p12d9f) basis set of Dyall [31] that was converted via the contraction scheme (222231211111111/5311111111111/42111111/42111) to a [15s13p8d5f] contracted basis. The contraction was carried out to minimize basis set superposition errors (BSSE), which was tested by assessing the BSSE via the counterpoise method [32]. The BSSE of calculated HgA (^2^Σ ^+^) and HgA^+^ (^1^Σ ^+^) molecules was found to be smaller than 0.5 kcal/mol. The [15s13p8d5f] basis set is of VDZ quality in the core region, however of VTZ quality in the valences space. Therefore it was combined with Dunning aug-cc-pVTZ basis sets for Li and Na [33]. For K, a 6–311++G(3df) basis was employed [34] whereas for Rb a (21s15p9d3f)[14s10p5d1f] basis set was used [31]. When calculating molecules composed of elements from different periods of the periodic table, a balanced choice of basis sets is essential for obtaining molecular properties of comparable accuracy. We tested the compatibility of the basis sets chosen by calculating ionization potentials (IPs) of the various atoms and comparing them with experimental IPs (Table 1). Errors in NESC/CCSD(T) IPs are 1.9 ± 0.9 % (smallest error for Li: 1 %; largest error for Hg: 2.7 %), which indicates a consistent description of all elements by the basis sets chosen.

In this work, only scalar relativistic corrections were considered. Spin-orbit coupling (SOC) effects can become large in magnitude for Z > 50 and when the fractional occupation of p- and d-orbitals is significant. Hence, SOC effects should definitely not play any role for the closed shell systems HgA^+^ (^1^Σ ^+^) and even for the open shell HgA (^2^Σ^+^) diatomics they should be rather small. In view of the fact that we are primarily interested in HgA bonding as reflected by the corresponding BDE values, we assume that SOC is a second order effect and discard it in this work. However, we have also to consider that we use molecular orbital (MO) theory to describe HgA bonding. It is a well-known fact that SOC leads to a change of the non-relativistic MOs, for example by mixing σ and π MOs. As we will show in the following, bonding in HgA is exclusively based on σ and σ* MOs so that a sizeable mixing in of π MOs via SOC is unlikely. In any case, trends in BDE values can be discussed in terms of scalar relativistic MOs as will be done in this work.

For the purpose of comparing calculated BDE values with experimental bond dissociation enthalpies at 298 K (BDH(298)), the former were converted to the later by calculating zero-point energies and thermal corrections, which was carried out using NESC/B3LYP vibrational frequencies. The electron density distribution was calculated at NESC/B3LYP and investigated utilizing the natural bond order (NBO) analysis [35]. In addition, bond critical points **r_c_** were determined with the help of the toplogical analysis [9]. The values of the electron density ρ(**r_c_**) and the energy density H(**r_c_**) at the critical point **r_c_** were used to identify the character of the bonding interactions according to the Cremer-Kraka criteria [10,36]. In addition, virial charges of the atoms were calculated for all molecules by integrating over atomic volumes [9]. Dipole moments for the cations were calculated with regard to the center of charge (defined by the atomic numbers). All calculations were carried out with the program packages COLOGNE08 [37] and Gaussian03 [38].

## 3. Results and Discussions

In Table 1, some properties of atoms Hg, H, and A (A = Li, Na, K, Rb) are summarized where H is included for reasons of comparison. Calculated BDE values of HgA (^2^Σ ^+^) and HgA^+^ (^1^Σ ^+^) molecules are summarized in Table 2 together with bond lengths, dipole moments, charge transfer values, and first ionization potentials (IP).

NESC/CCSD(T) BDE values for HgA (^2^Σ ^+^) are smaller than the corresponding B3LYP values (deviations up to 1.6 kcal/mol; even larger deviations are obtained for ZORA-GI/B3LYP values). Similar deviations are obtained for HgA^+^ (^1^Σ ^+^) cations (Table 2), which seems to indicate that NESC/B3LYP performs surprisingly well in these cases. Considering however errors in percentage of the magnitude of the experimental BDEs (see Section 4), it becomes obvious that NESC/CCSD(T) is more reliable than NESC/B3LYP.

Relativistic corrections are between 0.6 (HgRb) and -10.8 kcal/mol (HgH), i.e. the non-relativistic BDE can be smaller or larger than the relativistic one. For the corresponding cations, corrections are between 0.3 (HgRb^+^) and 21 kcal/mol (HgH^+^). The relativistic corrections can change the non-relativistic BDE value by more than 50 % thus confirming the necessity of scalar relativistic corrections. We conclude that both NESC and CCSD(T) methodology is necessary to obtain reliable BDE values. Therefore, we will discuss in the following predominantly NESC/CCSD(T) results.

The calculated BDE values of HgA are rather small (less than 30% of the BDE value of HgH, Table 1) and decrease from 3.0 (A = Li) to 0.8 kcal/mol (A = Rb) whereas the corresponding NESC/CCSD(T) interatomic distances increase from 3.06 to 4.42 Å (Table 2). The B3LYP distances are somewhat shorter than the CCSD(T) values, however surprisingly close to the latter deviating by just 3 – 9 %. Both sets of values are shorter (NESC/CCSD(T): 0.080 – 0.388 Å, Table 2) than the predicted van der Waals distances (sum of van der Waals radii, Table 1) and seem to indicate some weak covalent bonding. The fact that the B3LYP description of HgA is reasonable despite the notorious failure of DFT in the case of van der Waals complexes seems to support this conclusion. There are also other observations, which seem to speak against a description of HgA diatomics as pure van der Waals complexes.

**Figure 1.**
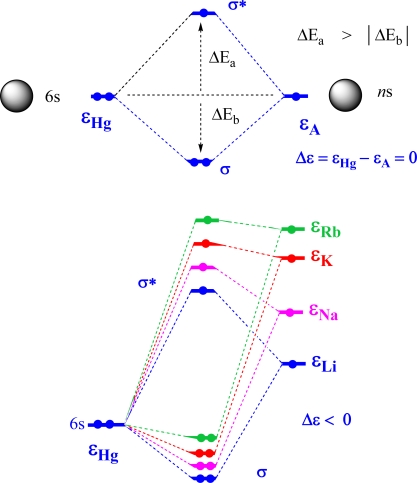
Orbital Schemes for Describing 3-Electron 2-Orbital Interactions. ^a^ ^a^ Top: AO interactions between two s-orbitals of equal energy ε. The stabilization energy |ΔE_b_| of the σ bonding MO is always smaller than the destabilization energy of the σ* antibonding MO. Bottom: Qualitative interaction diagrams of the AO 6s(Hg) with AOs *n*s(A) (*n* = 2, 3, 4, 5).

Comparable energies interact via orbital overlap and a stabilized bonding MO is formed as well as a destabilized antibonding MO [1–3]. For finite orbital overlap, the destabilization energy ΔE_a_ is always larger than the absolute value of the stabilization energy ΔE_b_ (Figure 1, top). The energy splitting between bonding and antibonding MO decreases with increasing AO energy difference Δε, i.e. covalent bonding is gradually converted into ionic bonding provided orbital overlap remains strong.

**Scheme 1.**
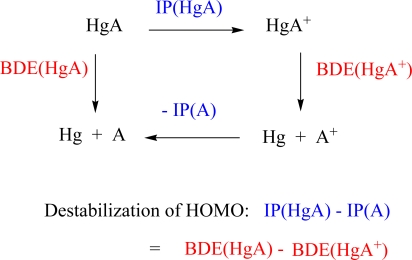
Thermodynamic Cycle to Determine the Destabilization Energy of the HOMO

Hg and A form a 2-orbital 3-electron system where the two AOs in question, 6s(Hg) and *n*s(A), form σ-bonding sub-HOMO and σ*-antibonding HOMO that are occupied by 3 electrons. Such an electron configuration is known to lead to some residual bonding. However, the bond strength strongly depends on the destabilization of the σ*-antibonding MO. If the AO energy difference is large (small), both ΔE_a_ and |ΔE_b_| will be small (large). The magnitude of Δε can be approximated by the difference of the first ionization potentials IP(Hg) – IP(A). Similarly, destabilization of the σ* MO can be assessed with the help of IP(HgA) referenced with regard to the first IP of A. The thermodynamic cycle of Scheme 1 reveals that the destabilization energy is exactly equal to the difference BDE(HgA) – BDE(HgA^+^).

In Figure 1 (bottom), the two highest occupied MOs of HgA are shown for the sequence A = Li, Na, K, Rb. In the case A = Li, the two AOs do not differ so much in size as one might expect from the atomic numbers of Li (3) and Hg (80). The diffuse character of the 2s AO leads to an atomic radius of 1.45 Å for Li whereas that for Hg is 1.50 Å (Table 1). Hence, there should be some overlap between 2s(Li) and 6s(Hg) AO. The energies of these AOs can be estimated from the IPs of the two atoms: 241 kcal/mol (10.44 eV) for Hg and 124 kcal/mol (5.39 eV) for Li (Table 1) [39], i.e. the 2s AO is located about 116 kcal/mol (5 eV) above the 6s AO so that the resulting σ MO is dominated by the 6s(Hg) AO and the σ* by the 2s(Li) AO. Hence, only a polar or ionic bond can result in this situation. This implies a charge transfer from the alkali to mercury atom in line with the lower electronegativity of A (0.79 < χ(A) < 0.98, Table 1) as compared to that of Hg (χ = 2.00; Pauling scale, Table 1).

There is however only the high lying 6p(Hg) AO as suitable acceptor orbital, which is difficult to populate. Accordingly, the calculated NBO charges transferred from A to Hg are just 22, 23, 42, and 30 melectron (Table 2), respectively. Hence, both ionic and covalent bonding are suppressed due to the electron configuration of Hg.

The BDE values of the cations HgA^+^ (^1^Σ ^+^) are 3 – 11 kcal/mol larger than those of the neutral molecules (Table 2). The largest value (13.8 kcal/mol) is obtained for HgLi^+^ (^1^Σ ^+^) whereas for the higher homologues values of 6.3, 5.1 and 4.4 are calculated (Table 2). The corresponding destabilization energies ΔE_a_ obtained according to Scheme 1 for the σ*MO of HgA are 10.8, 3.9, 4.1, and 3.6 kcal/mol. We can improve these values to 11.9, 6.9, 5.7, and 5.2 kcal/mol (Table 2) utilizing the experimental IPs (Table 1). The ΔE_a_ values indicate that the interaction of the 6s(Hg) and *n*s(A) AOs is weak because the large difference Δε (given by the difference IP(Hg) - IP(A) ≥ 111 or 116.4 kcal/mol, Table 1) suppresses strong covalent interactions and efficient 3-electron bonding in HgA. As a suitable reference, we have also calculated the BDEs of HgH (^2^Σ ^+^) and HgH^+^ (^1^Σ ^+^) and obtain values of 10.1 and 60.9 kcal/mol (Table 2), respectively. This implies that the σ*-antibonding MO is destabilized by ΔE_a_ = 50.8 kcal/mol thus reducing the covalent bond strength dramatically. Although the bond in the latter molecule is relatively weak, bonding can be considered as covalent with some polar (ionic) character. This is confirmed by the charge transfer from Hg to the more electronegative H atom (0.332 electron, Table 2).

Considering the data in Tables 1, 2, and Figure 1, one can see a relationship between the decreasing BDE values in the series HgLi to HgRb, the decrease in IP for A = Li to Rb, the raise in the orbital energy ε(*n*s), and the corresponding increase in Δε, which leads to a decrease in stabilizing interactions (Figure 1, bottom). For HgRb, orbital interactions are weaker than for HgLi and correspondingly the σ(HgRb) MO is less stabilized than the σ(HgLi) MO (Figure 1, bottom). Parallel to this trend, the destabilization energies of the σ* HOMO also decrease from HgLi to HgRb as shown in Figure 1 (see also Table 2).

The observed trends in the BDEs of diatomics HgA can be explained by orbital theory although the magnitude of the BDE values reminds of van der Waals complexes. For the purpose of clarifying the bonding situation, we analyzed the HgA bond density at the bond critical point (first order saddle point of the electron density distribution between the interacting atoms) applying the Cremer-Kraka criterion of covalent bonding [10,36]. A covalent bond is given when a path of maximum electron density with bond critical point connects the interacting atoms (necessary condition) and the energy density at the bond critical point is negative (sufficient condition), which indicates a stabilizing effect of the bond density [10,36]. As can be seen from the data in Table 3, HgH has at the bond critical point a bond density of 0.68 electron/Å^3^ and an energy density of −0.33 hartree/Å^3^ typical of a weak covalent bond. For HgH^+^, covalent bonding is even more pronounced as reflected by ρ(**r**_c_) and H(**r**_c_) of Table 3. In contrast to these bonds, the Hg-A and Hg-A^+^ interactions are ionic or van der Waals interactions as revealed by relatively small bond densities and positive energy densities at the critical point between the atoms. Since ionic bonding has already been excluded, HgA and HgA+ diatomics must be considered as van der Waals complexes.

In view of these results it has to be clarified why the BDE values of the HgA diatomics do not follow the increase in the polarizability of A from Li to Rb (Table 1) and why removing an electron does not lead to much stronger bonding in the resulting HgA^+^ molecules. The first question can be answered by considering the interaction distance between Hg and A. This is 2 – 10% shorter than the sum of van der Waals radii (Tables 1 and 2), which is typical of many non-bonded interactions when compared with van der Waals dimensions derived from crystal data. Hence, this shortening does not necessarily indicate any covalent bonding. Using the experimental polarizabilities of Hg and A (Table 1) and the calculated distances to estimate bond energies according to equation (1)

(1) $$\text{Δ}\text{E}={\text{constant}\;\text{α}(\text{Hg})\;\text{α}(\text{A})/\text{R}(\text{HgA})}^{6}$$

interaction energies result that decrease rather than increase with increasing atomic number of A thus confirming the quantum chemical calculations.

In the case of the cations there is the interaction between two spherical closed shell species. The positive charge of the molecule is preferentially localized at the alkali atom (more than 90%, Table 2), which due to its positive charge can polarize the density of Hg thus leading to electrostatic interactions. Li possesses the strongest polarizing power whereas for the larger atoms K and Rb positive charge is distributed over a larger volume and therefore their polarizing power is reduced. Because of this and the larger interaction distance, interactions become weaker with increasing atomic number. Hence both HgA (^2^Σ ^+^) and HgA^+^(^1^Σ ^+^) should be described as pure van der Waals systems hold together by dispersion and electrostatic forces and weakened by exchange repulsion.

The calculated virial charges (Table 3) are considerably larger than the NBO charges, which is a result of the fact that no longer an internal reference is used for the charge calculation as done for Mulliken, NBO, and other population analyses. Therefore, they are not suitable to determine the ionic character of the HgA bonds. For this purpose, one would have to take as a reference the “charge transfer” calculated for the superimposed, non-interacting atomic densities, which according to some test calculations adopts significant values. Nevertheless, the virial charges confirm the overall picture. They decrease from 306 melectron (Li) to 138 melectron (Rb, Table 3), which is in line with the increase in energy for the *n*s(A) AO and the corresponding reduced interaction between 6s(Hg) and ns(A).

We conclude that the charge transfer from Hg to A is an indicator for the onset of a weak covalent / ionic interaction in the case of HgA that is largely annihilated by the single electron occupation of the antibonding MO. Bonding is further weakened by a reduced tendency of orbital interactions with increasing atomic number of A due to an increase in Δε and reduced overlap. Although the HgA and HgA^+^ molcules clearly belong to the class of van der Waals complexes, we describe them as *orbital driven van der Waals complexes* that can be best understood by analyzing them as 3-electron 2-orbital systems with an onset of covalent character.

## 4. Comparison with Experimental HgA Data

Mercury-alkali compounds are potential candidates for excimer laser action and therefore all HgA systems described in this work have been investigated by experimental means. HgLi and its potential energy curve were studied by molecular beam scattering experiments [40–43], laser spectroscopy [44–48], and quantum chemical calculations [19,49]. Similar investigations were carried out for HgNa (molecular beam scattering experiments [50,52,53], pseudopotential calculations [19,54], and relativistic all-electron calculations [55,56]) and HgK (molecular beam scattering experiments, [51,57,58] pseudopotential calculations [59]). No experimental data are available for the corresponding cations. The results of the BDE measurements for HgA molecules (A = Li, Na, K, Rb) are summarized by Herzberg and Huber [22] and are compared in Table 4 with the NESC/CCSD(T) values obtained in this work. For this purpose, all BDE values were converted into BDH(298) = D_0_(298) enthalpies with the help of NESC/B3LYP frequencies.

Measured and calculated BDH(298) values for the HgA molecules are in reasonable agreement differing at the most by 0.9 kcal/mol with the exception of HgH^+^, for which however the experimental value is uncertain [22,60]. In view of this agreement, one can assume that BDE and BDH values for the corresponding cations (Tables 2 and 4) are also reliable. We note in this connection that an extensive pseudopotential study of the potential energy curves of HgA used cc-pVQZ basis sets with several sets of diffuse functions and obtained a BDE value for HgLi that was 20% off the experimental value. The authors had to readjust the parameters of the pseudopotential to reproduce the experimental value [19]. Relativistic all-electron theory used in this work performs better in this respect, which is reflected by the fact that reasonable results are obtained even with the smaller cc-pVTZ basis set.

Two experimentally based Hg-Li distances have been reported (3.000 Å [40], 3.037 Å [47]), which are somewhat smaller than the value of 3.056 Å (Table 2) calculated in this work. Interaction distances have also been reported for HgNa and HgK (4.72 and 4.911 Å [40]), however these values are 0.7 Å longer than the calculated ones and therefore highly unlikely. These deviations reflect the fact that the distance was indirectly determined. The authors used the results of molecular beam experiments (measured differential cross sections and total cross sections) to derive the interaction potentials of HgNa and HgK, which then led to the interaction distances. This procedure however suffered from low signal-to-noise ratios, which obscured measured scattering data so that the minimum of the HgA potentials could not be derived accurately [19,40].

## 5. Chemical Relevance of Results

Although the primary objective of this work is the understanding of mercury-alkali interactions and a discussion of the properties of HgA and HgA^+^ molecules based on this understanding, we will investigate in this section to which extent the results obtained shed a light on the physics and chemistry of mercury-alkali metal systems in general. Alkali metals dissolve in liquid mercury and form alkali metal amalgams. Best known is the sodium amalgam used in the chlor-alkali process to produce sodium hydroxide. The amalgam formed at the mercury cathode in the electrolysis of aqueous sodium chloride is continuously removed from the process and reacted with water, which decomposes the amalgam into sodium hydroxide and mercury. Wastewaters of chlor-alkali plants are contaminated by mercury and are one of the major reasons for mercury contamination of the environment [61].

Alkali metal amalgams have become interesting for technological purposes because of their unusual electronic and thermodynamic properties. Many of them are liquid (for lower concentrations of alkali metal in Hg) and change their thermoelectric power, viscosity, mixing entropy, and electrical resistivity in dependence of the percentage of alkali metal in characteristic and unexpected ways [62–65]. For example, the electrical resistivity of an alkali metal amalgam adopts its maxium at 60 % [65]. Investigations of liquid HgA alloys using EXAFS (Extended X-ray Absorption Fine Structure) [63,64], XANES (X-ray Absorption Near-Edge Spectroscopy) [64], and neutron diffraction methods [62] suggest that Hg-polyanions are formed containing 4 or 5 Hg atoms, which surround an alkali atom. This is in line with observations made for Hg-alkali crystalline amalgams [66,67] that reveal a tendency to form clathrate-type structures composed of Hg_4_ structural units that encage alkali elements.

The properties calculated for the HgA diatomics in this work support and rationalize these observations. Because of the weak interactions between Hg and A, the alkali metal can dissolve in liquid Hg where at lower A-concentrations each A atom is probably surrounded by a solvation shell of Hg atoms formed by electrostatic interactions. With an increasing number of A atoms local order seems to develop, which is charge transfer driven. According to the calculated virial charges of Table 3, each A atom (A > Li) can donate negative charge to 4 to 7 surrounding Hg atoms. Space limitations resulting from exchange repulsion between pairs of Hg atoms will reduce this number to 4 thus leading to arrangements of Hg_4_^−^ A^+^ ion pairs where a square or tetrahedral arrangement of the Hg atoms is in principal possible.

According to EXAFS and neutron diffraction experiments, the Rb-Hg distance is 3.60 up to 3.66 Å for the liquid RbHg alloy [62,63], which is similar to the shortest Rb-Hg distance observed in the crystalline Rb_5_Hg_19_ structure [66]. This is close to the Rb-Hg cation distance of 3.73 Å calculated in this work (Table 2) and an indirect confirmation for the presence of ionized alkali atoms in connection with Zintl-type Hg_4_ units carrying negative charge. These units and an overall order in the liquid alloy at A concentrations beyond 20 at. % seem to be responsible for the unusual electrical conductivity and other properties of liquid alkali amalgams.

## 6. Conclusions

NESC/CCSD(T) all-electron calculations with augmented VTZ basis sets lead to reasonable descriptions of HgA and HgA^+^ diatomics (A = Li, Na, K, Rb), their BDEs, interaction distances, and charge distributions. Calculated and experimental BDH(298) values are in reasonable agreement with deviations ≤ 1 kcal/mol. The analysis of the BDEs reveals that relativistic corrections are as large as 100% of the bond strength and therefore decide on the accuracy of the computed values. Calculated relativistic bond lengths are 2 – 10% smaller than ideal van der Waals distances, which is typical of many van der Waals complexes. This is confirmed by the investigation of the electron density distribution applying the Cremer-Kraka criteria of covalent bonding. Future studies will have to verify the structure of the electron density in atom and interatomic reasons using, for example, electron localization functions as described by Silvi and Savin [68] and recently by Putz [69].

Trends in the BDE values of HgA and HgA^+^ can be explained via a 3-electron 2-orbital model normally used in the case of covalent bonding, which causes us to speak of *orbital-driven van der Waals complexes*. The model used is based on a comparison of first ionization potentials of HgA and A (or Hg) and BDE values of HgA and HgA^+^ according to the thermodynamic cycle shown in Scheme 1. It is generally applicable to other HgX bonding situations [21]. The results obtained for the HgA and HgA^+^ diatomics are useful to discuss the formation of Zintl-type Hg_4_ units with negative charge surrounding A^+^ in liquid alkali metal amalgams. For A = Rb, the calculated Rb-Hg distance of the cation (3.7 Å, Table 2) is similar to the Rb-Hg distance found with EXAFS and neutron diffraction methods (3.6 and 3.66 Å [62,63]) thus supporting the ion pair model.

## Figures and Tables

**Table 1.** Properties of Hg, H, and alkali atoms.^a^

Atom (State)	Pauling χ	Allred Rochow χ	IP NESC/CCSD(T) [kcal/mol]	IP exp [kcal/mol]	Atomic, Covalent Radius Å	vdW radius Å	Polarizability α [Å^3^]
H(^2^S)	2.20	2.20	313.5^b^	313.6	0.25, 0.37 (1.86)	1.20 (2.75)	0.67
Li(^2^S)	0.98	0.97	123.2	124.3	1.45, 1.34 (2.83)	1.82 (3.37)	24.3
Na (^2^S)	0.93	1.01	115.6	118.5	1.80, 1.54 (3.03)	2.27 (3.82)	23.8
K(^2^S)	0.82	0.91	98.5	100.1	2.20, 1.96 (3.45)	2.75 (4.30)	43.4
Rb(^2^S)	0.82	0.89	94.6	96.3	2.35, 2.11 (3.600)	2.95 (4.50)	47.3
Hg(^1^S)	2.00	1.44	234.2	240.7	1.50, 1.49 2.98	1.55 (3.10)	5.7

a Pauling and Allred-Rochow electronegativities χ from Ref. 14, first ionization potentials IP from NESC/CCSD(T) calculations or experiment [39], atomic radius, covalent radius, and van der Waals (vdW) radius from Ref. [14], polarizability from Ref. [39]. Values in parentheses give the ideal covalent HgA (HgH, HgHg) bond length estimated from covalent radii and the ideal HgA (HgH, HgHg) van der Waals distance estimated from van der Waals radii.

b Calculated from the atomic energy.

**Table 2.** Calculated bond lengths *R*, bond dissociation energies BDE, NBO charges *q*, dipole moments, and ionization potentials *IP* for HgA (^2^Σ ^+^) and HgA^+^ (^1^Σ ^+^) molecules. a

Molecule (State)	R(HgA) NESC/B3LYP [Å]	BDE NESC/B3LYP [kcal/mol]	R(HgA) NESC/CCSD(T) [Å]	BDE NESC/CCSD(T) [kcal/mol]	q(Hg) [electron]	Dipole Moment [Debye]	IP [kcal/mol]
HgH (^2^Σ^+^)	1.784	11.7	1.749	10.1 (10.0)	0.332	0.38	183.3
HgLi (^2^Σ^+^)	2.917	4.4	3.056	3.0 (2.9)	−0.022	0.28	112.4
HgNa (^2^Σ^+^)	3.333	3.0	3.432	2.4 (2.3)	−0.023	0.47	111.6
HgK (^2^Σ^+^)	3.830	2.0	4.197	0.9 (0.8)	−0.042	0.58	94.4
HgRb (^2^Σ^+^)	4.052	0.7	4.417	0.8 (0.7)	−0.030	0.64	91.1
							**Destab ΔE_a_**
HgH^+^(^1^Σ^+^)	1.606	63.2	1.597	60.9 (60.9)	0.959	0.30	50.8, 57.4
HgLi^+^(^1^Σ^+^)	2.674	14.9	2.709	13.8 (13.8)	0.090	9.18	10.8, 11.9
HgNa^+^(^1^Σ^+^)	3.031	10.1	3.097	6.3 (6.2)	0.070	10.36	3.9, 6.9
IIgK^+^(^1^Σ^+^)	3.521	5.2	3.551	5.1 (5.0)	0.029	11.54	4.1, 5.7
HgRb^+^(^1^Σ^+^)	3.726	3.3	3.735	4.4 (4.4)	0.021	10.31	3.6, 5.2

a BDE values in parentheses give NESC/CCSD(T) results obtained at NESC/B3LYP bond lengths. Since the potential is very flat, they do not deviate from BDE values obtained for optimized bond lengths. – Dipole moments are oriented from Hg (negative end) to A (positive end, physical notation). For the cations, the dipole moment was determined with regard to the center of charge as determined by the atomic numbers of Hg and A. – NBO charges *q* are given for the Hg atom. – Destab ΔE_a_ gives the destabilization of the σ* MO determined according to the thermodynamic cycle of Scheme 1 where the first entry is derived exclusively from calculated IPs and the second uses also the experimental IP(A) and IP(Hg) values of Table 1.

**Table 3.** Analysis of the Bond Density. a

Molecule	Bond density ρ(r_c_) [e/Å^3^]	Energy density H(r_c_) [hartree/Å^3^]	Position of r_c_ Δ(Hg) [%]	Atomic Charge Q(Hg) [melectron]
HgH (^2^Σ^+^)	0.681	−0.331	−34.8	387
HgLi (^2^Σ^+^)	0.069	0.004	37.7	−306
HgNa (^2^Σ^+^)	0.053	0.002	22.9	−218
HgK (^2^Σ^+^)	0.037	0.002	10.7	−158
HgRb (^2^Σ^+^)	0.033	0.002	5.9	−138
HgH^+^ (^1^Σ^+^)	1.004	−0.751	−42.2	786
HgLi^+^ (^1^Σ^+^)	0.099	0.006	36.4	43
HgNa^+^ (^1^Σ^+^)	0.074	0.011	22.9	38
HgK^+^ (^1^Σ^+^)	0.053	0.006	10.7	33
HgRb^+^ (^1^Σ^+^)	0.048	0.004	6.6	30

a The bond density is represented by the density ρ(**r**) at the bond critical point, **r**_c_. The position of **r**_c_ is measured by the shift Δ (given in %) with regard to the midpoint of the interaction distance HgH or HgA. Positive Δ indicate a shift toward A. Atomic charges Q(Hg) are calculated with the virial partitioning method [9].

**Table 4.** Comparison of Experimental and Theoretical Bond Dissociation Enthalpies BDH(298). a

Molecule	Exp. BDH(298) [kcal/mol]	NESC/CCSD(T) BDH(298) [kcal/mol]	NESC/B3LYP Frequncy [cm^−1^]
HgH (^2^Σ^+^)	9.5	9.2	1245
HgLi (^2^Σ^+^)	3.3	3.3	167
HgNa (^2^Σ^+^)	2.2	2.7	72
HgK (^2^Σ^+^)	1.97±0.05	1.2	47
HgRb (^2^Σ^+^)	2.0	1.1	32
HgH^+^ (^1^Σ^+^)	50 – 69	58.9	1997
HgLi^+^ (^1^Σ^+^)		14.0	262
HgNa^+^ (^1^Σ^+^)		6.6	123
HgK^+^ (^1^Σ^+^)		5.4	67
HgRb^+^ (^1^Σ^+^)		4.7	45

a Experimental bond dissociation enthalpies BDH(298) = D_0_(298) have been taken from Ref. 22. Vibrational and thermal corrections of calculated BDEs are based on NESC/B3LYP frequencies.
